# Chemically and thermally stable silica nanowires with a β-sheet peptide core for bionanotechnology

**DOI:** 10.1186/s12951-016-0231-8

**Published:** 2016-12-01

**Authors:** Zahraa S. Al-Garawi, George E. Kostakis, Louise C. Serpell

**Affiliations:** 1 School of Life Sciences, University of Sussex, East Sussex, Falmer, BN1 9QG UK; 2 Chemistry Department, College of Sciences, Al-Mustansyria University, Baghdad, Iraq

**Keywords:** Amyloid fibril, Chemical stability, Thermal stability, Nanostructures, FTIR, Ultra microtome, TGA analysis

## Abstract

**Background:**

A series of amyloidogenic peptides based on the sequence KFFEAAAKKFFE template the silica precursor, tetraethyl orthosilicate to form silica-nanowires containing a cross-β peptide core.

**Results:**

Investigation of the stability of these fibres reveals that the silica layers protect the silica-nanowires allowing them to maintain their shape and physical and chemical properties after incubation with organic solvents such as 2-propanol, ethanol, and acetonitrile, as well as in a strong acidic solution at pH 1.5. Furthermore, these nanowires were thermally stable in an aqueous solution when heated up to 70 °C, and upon autoclaving. They also preserved their conformation following incubation up to 4 weeks under these harsh conditions, and showed exceptionally high physical stability up to 1000 °C after ageing for 12 months. We show that they maintain their β-sheet peptide core even after harsh treatment by confirming the β-sheet content using Fourier transform infrared spectra. The silica nanowires show significantly higher chemical and thermal stability compared to the unsiliconised fibrils.

**Conclusions:**

The notable chemical and thermal stability of these silica nanowires points to their potential for use in microelectromechanics processes or fabrication for nanotechnological devices.

**Electronic supplementary material:**

The online version of this article (doi:10.1186/s12951-016-0231-8) contains supplementary material, which is available to authorized users.

## Background

Highly organised, stable amyloid fibrils can be formed by a multitude of different proteins and peptides with a range of primary sequences. These fibrillar structures share a cross-β core structure, stabilised by multiple hydrogen bonds to produce degradation resistant fibrous structures. Whilst these amyloid fibrils play a significant role in a range of diseases known as the amyloidoses, they have been shown to have an important role in natural scaffolding materials such as insect silks, and therefore can be exploited for development of bionanomaterials [1]. Furthermore, the development of silica-nanowires templated by amyloid fibrils is a new, promising approach in bionanotechnology applications [2]. Biomineralisation has inspired researchers to create silica nanostructures by applying the sol–gel reaction of silica using one of the silica precursors; tetraethyl orthosilicate (TEOS) [2–6]. This reaction involves two steps; hydrolysis and condensation, resulting in a network of Si–O–Si chemical linkages. The core molecules can be nanospherical-particles [7], nanorods [8], nanofibers [9], or nanowires [10]. Moreover, amyloid-like fibrils have recently been used to form nanowires to imitate biomineralization behavior [6, 11]. These silica nanomaterials have potential in a wide range of applications such as electronics, catalysis, and sensors as well as drug delivery [12, 13]. Additionally, peptide nanowires have recently been used as part of electrochemical and medical biosensing [14, 15].

We previously characterized amyloid fibril structure of an amyloidogenic 12 residue peptide KFFEAAAKKFFE [16, 17] revealing an architecture in which the cross-β structure is stabilized via π–π interactions and electrostatic interactions. In order to investigate what drives assembly, we introduced substitutions of lysine to alanine (K/A) and lysine to arginine (K/R) and revealed that K plays an essential role in lateral association whilst substitution of the larger R residue led to increased β-sheet distance [17]. In recent work, we utilized this range of K > A or K > R substituted variants and showed that these are able to form silica nanowire structures (silica-NWs) via hydrolysis of the silica precursor TEOS, with varying degrees of efficiency and resulting in nanowires with different morphologies [11]. We showed that the position of the K residues in the sequences was important in determining the efficiency of silica-templating [11]. The inherent instability of many proteins under thermal and chemical conditions makes them incompatible with durable techniques [18]. Here we compare the characteristics of the original amyloid fibrils with the resulting silica-NWs (monomeric Si–OH, complexes Si–OR, or colloidal Si–O–Si), and monitor these structures to demonstrate the chemical and thermal stability of these supramolecular assemblies under a range of highly denaturing conditions using transmission electron microscopy (TEM), thermogravimetric analysis (TGA) and Fourier transform infra-red spectroscopy (FTIR).

## Methods

### Peptide preparation

Synthetic peptides prepared using FMOC synthesis (95% purity) were a gift from Dr. Helen Walden (Cancer research UK) [17]. Variants of NH_2_-KFFEAAAKKFFE-COOH shown in Table 1 were prepared in either water or phosphate buffered saline (PBS) and allowed to self-assemble into mature amyloid fibrils, which were then examined immediately (t0) or after 7 days incubation at room temperature (RT).

**Table 1:** Peptide sequences and buffers used for preparation

Sequence	Abbreviation	Solvent
**A**FFEAAAKKFFE	K1A	PBS
KFFEAAA**A**KFFE	K8A	H_2_0
**R**FFEAAAKKFFE	K1R	PBS
**R**FFEAAA**R**KFFE	K1RK8R	PBS
KFFEAAA**R**KFFE	K8R	PBS
KFFEAAAK**R**FFE	K9R	PBS

### Preparation of silica nanowires (silica-NWs)

Silica-NWs were prepared as previously described [11]. Briefly the reaction was carried out by hydrolyzing a cold solution of 2 mL TEOS (final concentration of 2 mM) with 500 μL of the fibrillized peptide solution (5 mg/mL of mature fibrils in 50 mM Tris–HCl buffer, pH 6.8), vortexed vigorously for 1 min, and a white precipitate was collected after 36 h at RT. The resulting white precipitate was washed with annular ethanol 99% × 3, centrifuged at 14,000 rpm for 3 min and then dissolved by milli-Q water for stability assays.

### Transmission electron microscopy (TEM)

Four microlitre peptide sample was placed onto 400 mesh copper TEM grid with Carbon/Formvar film (Agar Scientific) for 2 min, followed by a negative staining with 2% uranyl acetate for another 2 min. The grid was blotted after each incubation time using filter paper. Grids were examined using a Hitachi 7100 electron microscope operated at 80 kV and images collected on a Gatan Ultrascan 1000 (2K × 2K pixel) CCD camera (Gatan, Inc., Pleasanton, USA).

### Fourier transform infrared spectroscopy (FTIR)

Powder samples of silica-NWs were produced by washing the white precipitates with ethanol and then drying under N_2(g)_. Powder samples of variants after silica coating and after treatment (as described below) were then placed into the sample holder of FTIR-ATR instrument (Perkin Elmer Spectrum one), inserted into the photoacoustic cell and then sealed for FTIR measurement. Air was used as a control.

### Ultra Microtome thin sectioning and electron microscopy of silica-NWs

After 5 months incubation at room temperature, dry samples of selected silica-NWs were incubated in TAAB low viscosity resin (TAAB Laboratories Equipment Ltd, Aldermaston, UK). They were left for 24 h at room temperature to infiltrate with the resin before polymerizing overnight at 60 °C. Thin (c. 90 nm) sections were cut on a Leica Ultracut ultramicrotome (Leica Microsystems [UK] Ltd., Milton Keynes, UK), collected upon TEM support grids and stained with 0.5% (w/v) uranyl acetate for 1 h. Thin sections were examined using a Hitachi 7100 TEM operated at 100 kV accelerating voltage, and digital images were acquired with a Gatan Ultrascan 1000 (2K × 2K pixel) CCD camera (Gatan, Inc., Pleasanton, USA).

### Chemical and thermal stability fibrils before and after silica coating

#### Chemical stability

A solution of fibrils before and after silica coating (50 μL) was mixed with 50 μL of the following organic solvents; 2-propanol, annular ethanol 99%, acetonitrile (AcN), or with PBS-HCl, pH 1.5 and incubated for 1 h; and then imaged by TEM. These peptides were incubated with these solvents for up to 4 weeks and then characterised further by TEM and FTIR [19].

#### Thermal stability

A stock of silica-NWs solution (5 mg/mL) was diluted to a final concentration of 2 mg/mL, and incubated in a water bath at 70 °C for 1 h, and then used for further characterisation. Alternatively, the stock was diluted to a final concentration of 2 mg/mL, and autoclaved at 121 °C, 2 atm, and then used for further characterisation. These two experiments were also performed on solutions of fibrils before silica coating to run them as controls.

#### Stability over time

To study the chemical stability of silica-NWs over time, they were incubated with organic solvents or at low pH for 12 months and further characterised.

### Thermogravimetric analysis (TGA)

The thermal stability of silica-NWs after 12 months incubation was studied using TGA. A stock of 5 mg of each silica-NW sample was loaded to TGA machine (Q50, TA), and then heated up to 1000 °C, under N_2_, at 20 °C/min heating rate for around 4 h per sample. Q-series and TA-Universal analysis were used to analyse the percentage of change of weight relative to the temperature.

## Results and discussion

Fibres before and after silica coating have been well characterised previously using TEM, XRFD, FTIR, and TEM [11]. All the variant peptides examined formed amyloid structures before silica coating although their lateral association and morphology differed with sequence. Some, but not all the fibrils could be coated to form silica-NWs with cross-β core, despite the harsh conditions required for silica coating. The retention of the peptide core was previously confirmed by β-sheet spectra by FTIR and cross-β patterns from XRFD [11].

### Chemical and thermal stability of peptide fibrils versus silica-NWs

We sought to investigate the thermal and chemical structural stability of K/A, and K/R before and after siliconisation to gain insights into their physical properties for potential integration into nanotechnological devices and assemblies.

**Fig. 1:**
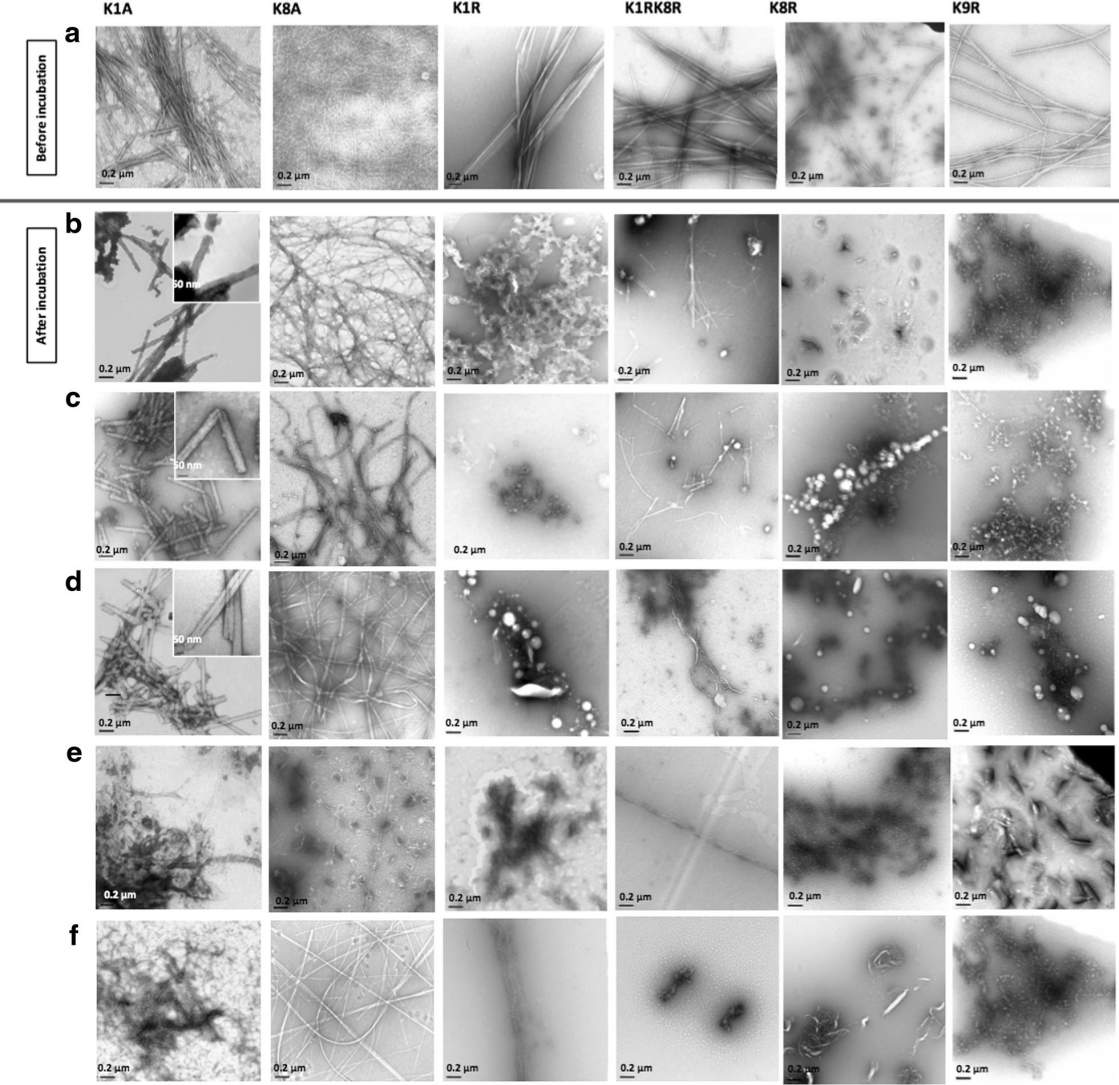
TEM images showing the chemical and thermal stability of K/A and K/R fibrils before (**a**) and after incubation for 1 h with harsh conditions. These harsh conditions included (**b**) 2-propanol, **c** ethanol, **d** AcN, **e** HCl, pH 1.5, and **f** heating up to 70°. K/A variants were initially stable when incubated with organic polar solvents but not at low pH. Moreover, K1A peptide was able to form nano-tubular structures in organic solvents. K/A variants were initially stable when heated up to 70 °C, while K/R variants underwent degradation into small non-fibrillar molecules following incubation in all conditions

The stability of the original peptide fibrils, non-silica coated fibrils were examined following harsh treatments with different solvents that are used for conventional lithography processes, including 2-propanol, ethanol, acetonitrile (AcN), and in a highly acidic environment (pH 1.5). The resulting structures are shown in electron micrographs in Fig. 1. All K/R fibrils were damaged when incubated with strong polar solvents or after heating and the resulting morphologies demonstrated amorphous aggregates. In contrast, we observed that fibrils formed by K/A variants were initially stable and maintained their original structures following heating or treatment with organic solvents highlighting the remarkable stability of these amyloid fibrils. K1A was able to withstand pH 1.5 whilst K8A was damaged in acid. Furthermore, K1A fibrils were also observed to undergo some morphological changes to form nano-tubular structures when treated with organic solvents (Table 2; Fig. 1b–d).

**Table 2:** Stability of non-silica coated K/A, and K/R fibrils in organic solvents, pH 1.5 and high temperature

Variants	Ethanol	2-propanol	AcN	pH 1.5	Autoclaving and heating to 70 °C
K1A	++++	++++	++++	+	−
K8A	+++	++	+++	−	+++
K1R	−	−	−	−	−
K1RK8R	−	−	−	−	−
K8R	−	−	−	−	−
K9R	−	−	−	−	−

+, sign used to denote stability following treatments; while −, signs refer to un-stable fibrils treated with harsh conditions

The resistance of K/A amyloid fibrils to the organic solvents is intriguing and two explanations are possible. Our previous work showed very close packing arrangement of the β-sheets excluding water [17]. This close interdigitation of the hydrophobic side chains may enable the fibrils to resist denaturation by solvents. Alternatively, it is possible that the interactions between the hydrophobic core (A^5^A^6^A^7^) and hydrogen bonds within-sheets are disrupted by the highly acidic or polar solvents, leading to a substantial rearrangement and resulting in altered fibrous architectures. Indeed, the structures shown in Fig. 1 do appear to be increasingly ordered. This may optimize favorable electrostatic interactions among the charged groups (E^4^, K^8^, and E^12^ in K1A, and K^1^, E^4^, K^9^, and E^12^ in K8A) [20]. In contrast, the K/R fibrils are unable to withstand denaturation by highly polar organic solvents, heating or low pH and this may arise from their more open conformation.

**Fig. 2:**
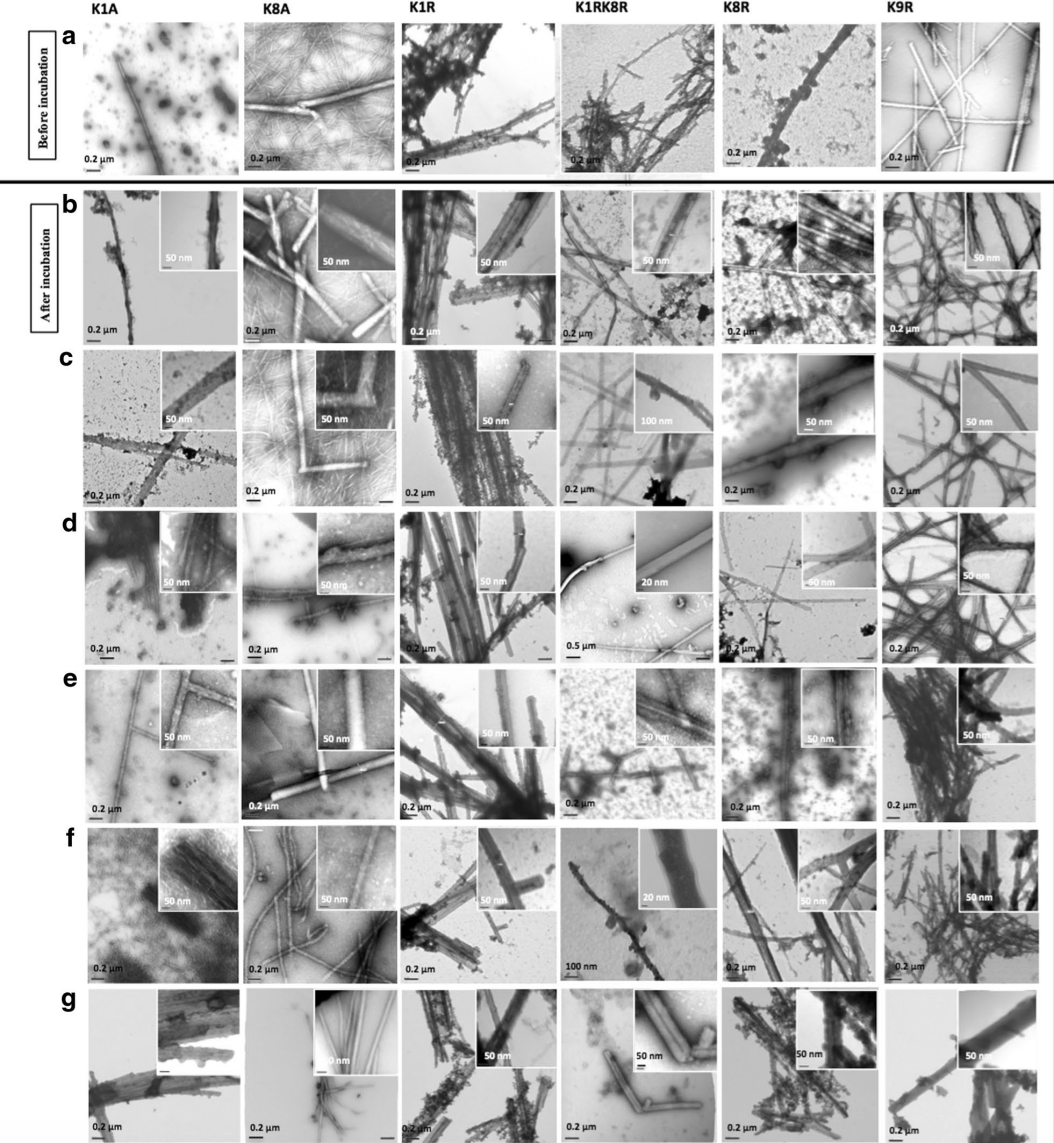
TEM images showing the ultrastructures of silica-NW before and following chemical and thermal treatment. **a** shows silica-NWs prior to treatment to compare to structures after incubation. Structures following incubation in **b** 2-propanol, **c** ethanol, **d** AcN, **e** HCl, pH 1.5, **f** heating up to 70 °C, and **g** autoclaving for 15 min, 2 atm, 1. All silica-NWs remained stable following incubation in harsh conditions

To compare the stability of the fibrils before and after silica coating, silica-NWs were treated in an identical way with solvents, low pH and high temperature. Figure 2 shows the corresponding TEM images of silica-NWs of K/A, and K/R following incubation under these conditions. There did not appear to be any major alterations in the morphologies and all the silica-NWs suprastructures remained. We have previously shown that very few silica-NWs are formed by K1A, whilst K8A, K1R, K1RK8R, K8R and K9R formed silica-NWs with a β-sheet peptide core [11]. Interestingly, although K1A forms silica-NWs very poorly on initial coating [11], after time and treatment, the formation of silica-NW appears to be enhanced (Fig. 2). After heating the silica-NWs up to 70 °C for 1 h and autoclaving, the morphology of these structures remained similar to prior to heating, showing that the silica-NWs were also thermally stable (Fig. 2f, g).

**Fig. 3:**
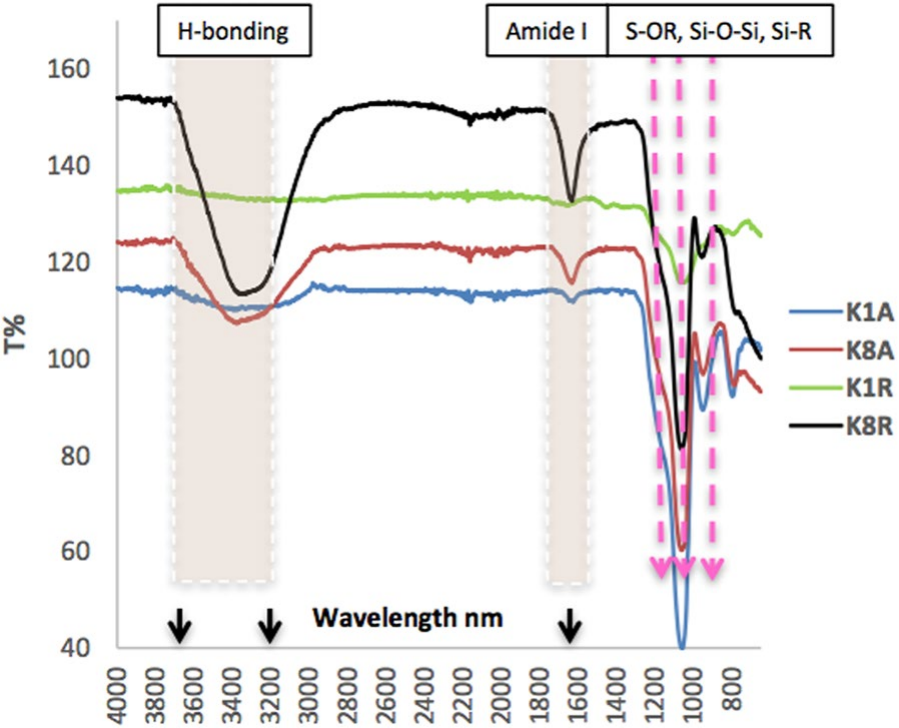
FTIR spectra of four silica-NWs autoclaved at 121 °C, 2 atm, for 15 min. All silica-NWs retained their β-sheet structure within siliconised architectures after autoclaving, as the presence of amide I bands indicates

FTIR spectra of two K/A and two K/R silica-NWs are shown in Fig. 3 following autoclaving. K8A and K8R showed very intense signals for H-bonding and all the silica-NWs showed strong signals for silica resonances. The silica-NWs except for K1R all showed Amide I bands following autoclaving supporting the existence of some residual β-sheet core structure. This high stability and robustness of silica-NWs indicates the strength of hydrophobic interaction combined with hydrogen bonding within the peptides [20].

### Chemical and thermal stability of silica-NWs after prolonged incubation

Further studies were carried out to examine the resistance of silica-NWs to the organic solvents, and heating after 1 month. The silica-NWs retain their structures and no major differences in morphology were observed after 1 month incubation (Additional file 1: Figure S1). Furthermore, these harsh environments did not prevent branching of silica-NWs of K/R and successful oxygen-bridging of Si^+4^ after a month incubation, which continue to polymerize and ultimately form multiple silica layers (Additional file 1: Figure S2). All silica-NWs were stable over a year although they appear to become thicker with glass-like morphology following incubation over 5 months, which may indicate the full oxygen-bridging of the tetrahedral silica structures (Q4) over the peptide core, as shown by sectioned TEM images (Additional file 1: Figure S3). The structure of silica peptide network is related to the polymerization of silica along the peptide surface template, then formation of tetrahedron structures (Q^n^ species). Usually, these species are connected to one another by bridging oxygen, and numbers of n ranging from zero (isolated tetrahedron) to 4 (fully oxygen bridging- 3D silica structures). Therefore, Q^3^ configuration, for example, refers to one NBO, and three bridging oxygen (BO), [21].

**Fig. 4:**
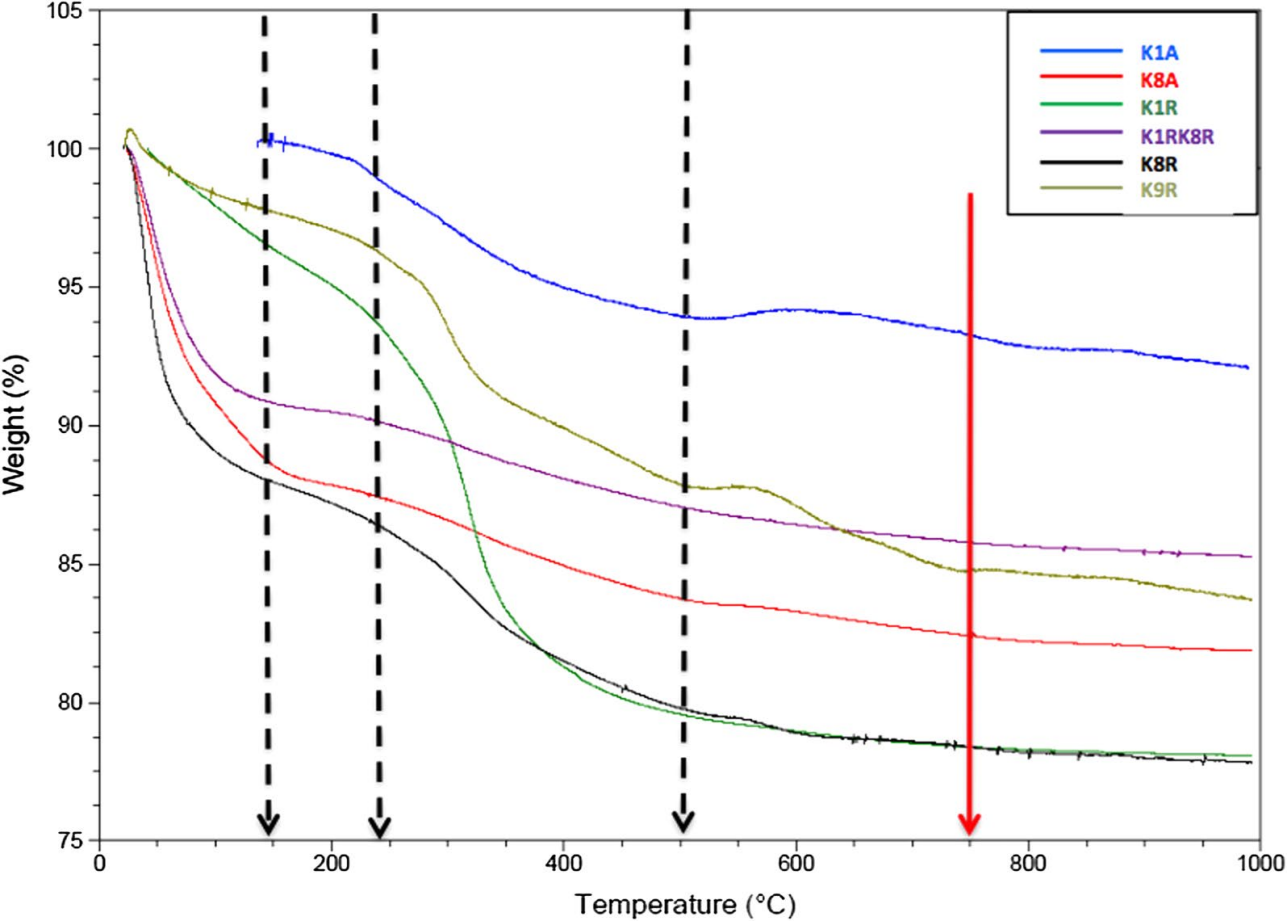
TGA analysis of silica-NWs (12 months old) showing the stability up to 1000 °C. For K/A S-NWs and K/R S-NWs the rate of the temperature increase was 20 °C/min, under N_2_

To determine the physical and chemical stability of silica-NWs after aging for 12 months, TGA analysis was carried out to monitor the weight loss as a function of increasing temperature up to 1000 °C, and FTIR spectra before and after heating were collected. The thermo-gram of silica-NWs shows a certain amount of weight decrease ranging from 2 to 14% up to 150 °C (Fig. 4; Table 3), presumably due to the evaporation of the solvent molecules that are accommodated within the silica-NWs. These results are consistent with the ordered silica-NWs having bound water molecules. The percentage of weight lost at temperatures ranging 150–500 °C was ~3–5% for K/A and 10–17% for K/R silica-NWs, except K1RK8R which lost only 3% weight when the temperature was raised from 150 to 500 °C. Above 500 °C the decrease progressed very slowly and gradually, and reached a low plateau at ~1000 °C. No major water loss was observed above 500 °C (~1–3% for K/A and 1–2% for K/R).

**Table 3:** Thermal stability of K/A, and K/R silica-NWs against heating up to 1000 °C, analysed by TA universal analysis software

K/A silica-NWs	Weight loss % (25–150 °C)	Weight loss % (150–500 °C)	Weight loss % (500–1000 °C)	Total loss % (25–1000 °C)
K1A	4	3	1	8
K8A	12	5	2	19
K1R	2.6	17.4	1.7	22
K1RK8R	9	3.7	1.7	14.4
K8R	3	18	2	23
K9R	2.3	10	1	13.3

The thermographs of K1A and K8A showed a small decrease in weight ~2.5–3% up to 150 °C, and reached a low plateau above 500 °C, ~1–1.5%. This finding may indicate that these structures are relatively more stable than others due to the high content of Q_4_ units cross-linked the Si- molecules rather than Si-Lys or Si-Arg. A much larger decrease in weight was observed between 150 and 500 °C for silica-NWs composed from K1R (~17%), and K8R (18%), This larger reduction may arise from the higher hydrophilic content of K/R silica-NWs. Above 500 °C, all K/R silica-NWs suffered a slight decomposition around 1–2%.

**Fig. 5:**
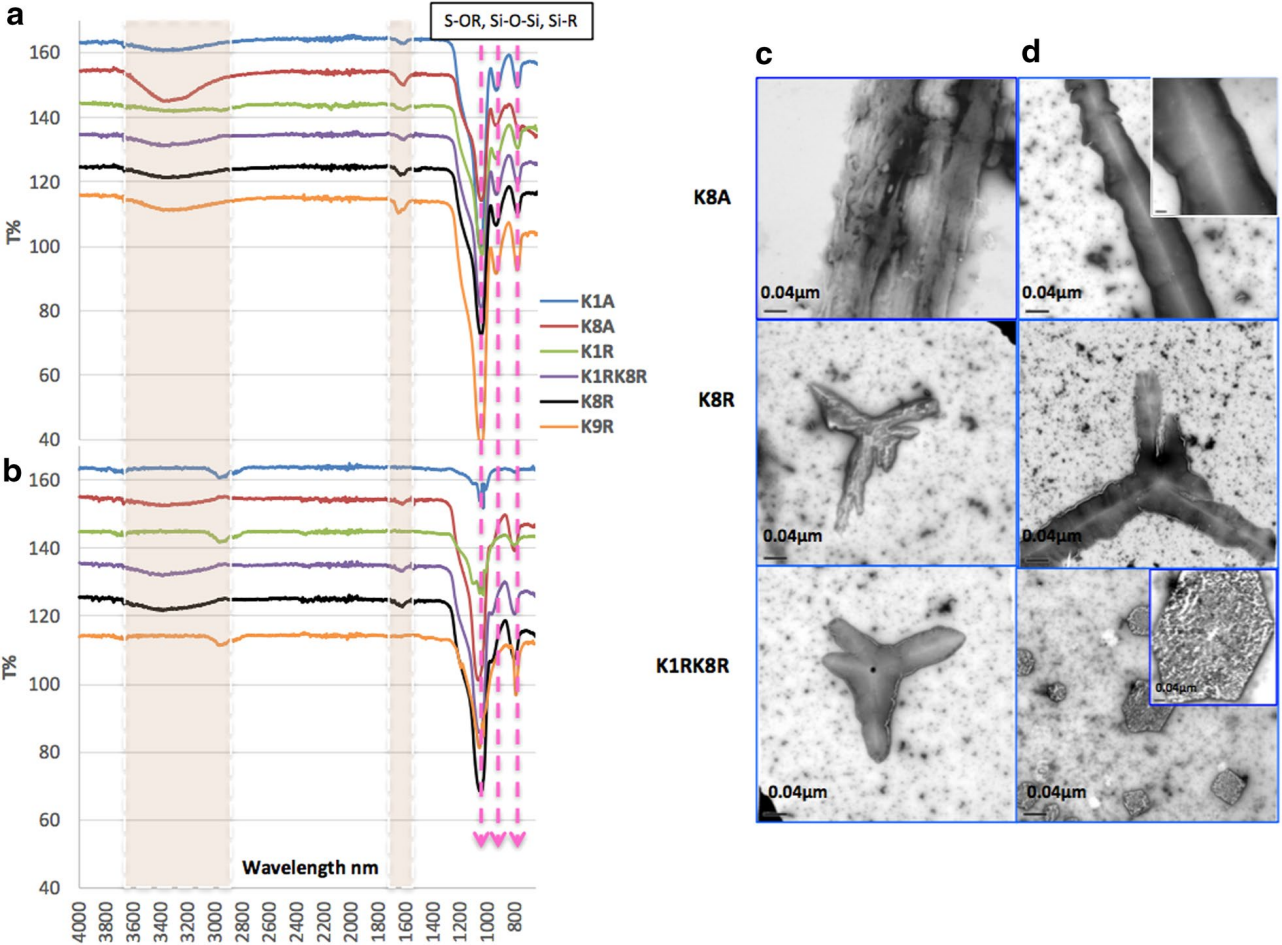
FTIR spectra of silica-NWs of K/A and K/R (12 months old) before and after heating up to 1000 °C. **a** before heating, **b** after heating for 1 h. Bands arising from H–bonding, and Amide I began to diminish, and some bands of Si–OR were visibly diminished after heating up to 1000 °C. Electron micrographs showing some examples of silica-NWs **c** before heating and **d** after heating, showing large glass like structures

All silica-NWs showed two-transformation states in weight loss %, except K1A, and K9R, which expressed three transition states at 150, 500, 750, up to 1000 °C. The loss of individual silica layers around the peptide, which refers to the Q_2_, and Q_3_ units, might cause the gradual decline observed in silica-NWs weight. This finding is consistent with FTIR spectra of silica-NWs before and after heating (Fig. 5).

FTIR spectra collected from the silica-NWs after 12 months pre-heating revealed symmetrical stretching vibrations of Si–O–Si, Si–OR and Si–O–Si bands (Fig. 5) [22, 23], along with bands arising from C–C, C=C, N–H, O–H and C=O bonds, which indicates that the silica-NW structure was retained. All silica-NWs also retained small Amide I band suggesting that they have maintained their peptide core. Peaks near 1050–950 cm^−1^ can be assigned to Si–O stretching vibration in Q^3^ units with one non-binding oxygen (NBO) per SiO_4_ tetrahedron, while peaks between 950–900 cm^−1^ can be assigned to Q^2^ units with two NBO (Fig. 5a, b) [24, 25]. Additionally, H–bonding bands were observed in the silica-NWs structures between 3664 and 3000 cm^−1^, in close agreement with C–H, O–H, Si–NH_2_, and amine N–H stretching, and NH_2_ vibration mode [26, 27], as well as Amide I, 1640–1613 cm^−1^, which represents β-sheet structures [28]. However, some peaks of Si–OR groups were removed by heating. The H–bonding bands remained post-heating up to 1000 °C, while some Amide I bands showed only weak signals. Silica-NWs formed from K1R and K9R appeared to lose their Amide I band, suggesting that they were unable to retain the core peptide structure under such extreme conditions. However, interestingly the other silica-NWs, K1A, K8A and K8R, did still show reduced Amide I bands. The extreme heating might cause more calcination of the silica-NWs, and therefore some of the organic content is removed [2], or it perhaps the unusually intense bands of Si-OR overcome the less intense bands of Amide I (Fig. 5b). This finding is supported by TEM micrographs in Fig. 5c and d, where thickened silica layers were replaced by large silica structures (Fig. 5d). The growth of silica layers polymerized on the inner and outer surfaces are affected by many factors. The total number of amino groups (surface charge density depending on the sequence of the peptide variant), has been suggested to be essential for regulating and controlling thicknesses and density of silica layers [4, 29, 30]. Together the results are consistent with the view that these silica-NWs are resistant to chemical and thermal attack. This stability may make the amyloid templated silica-NWs suitable for different applications requiring harsh processing conditions.

## Conclusions

The ordered silica-NWs used for this study were prepared previously utilising a self-assembling, positively charged peptide template. Previous TEM and XRFD, FTIR, and sectioning data showed that the silica-NWs retained cross-β peptide structures. Treatment of silica-NWs with harsh conditions clearly reinforces the exceptional conformational stability of these supramolecular fibrous structures even after prolonged incubation. This compatibility with harsh conditions indicates the potential for the incorporation of these silica-NWs into biosensors or other industrial applications necessitating chemical or thermal stability, such as conventional microelectronic mechanics processes for manufacturing of serviceable nano-technological machines.

## Additional file

**Additional file 1.** Additional figures.

## Abbreviations

Silica-NWs: silica nanowires
RT: room temperature
TEOS: tetraethyl orthosilicate
PBS: phosphate buffered saline
FTIR: Fourier transform infrared spectroscopy
UMTs: ultra microtome thin sectioning
AcN: acetonitrile
TGA: thermogravimetric analysis
