# Supplementary material for: Chemically and thermally stable silica nanowires with a β-sheet peptide core for bionanotechnology
Source: J Nanobiotechnology. 2016 Dec 1;14:79. doi: 10.1186/s12951-016-0231-8 (PMC5134108; doi:10.1186/s12951-016-0231-8)
Supplement: Supplementary file 1 — Additional file 1. Additional figures. [file 12951_2016_231_MOESM1_ESM.docx]

**Supplementary information**


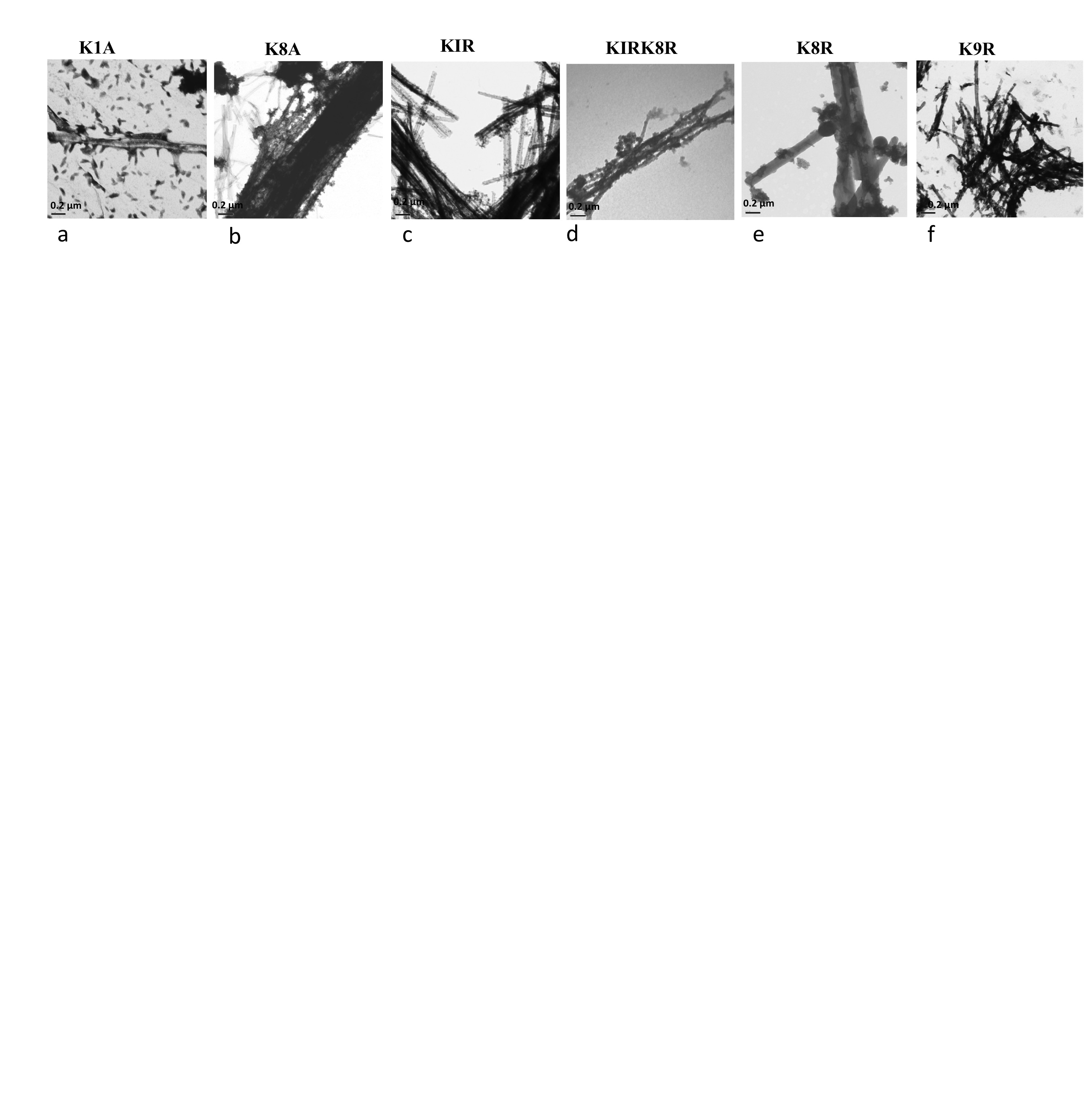


Figure S1. TEM images showing the stability of silica-NWs of K/A and K/R incubated under harsh conditions for one month with organic solvents such as a) 2-propanol, b) ethanol,, and c) AcN, d) acidic solution pH 1.5, and upon autoclaving (e), and heating up to 70 ˚C(f). All silica-NWs seemed able to maintain their structure after a month following harsh treatment


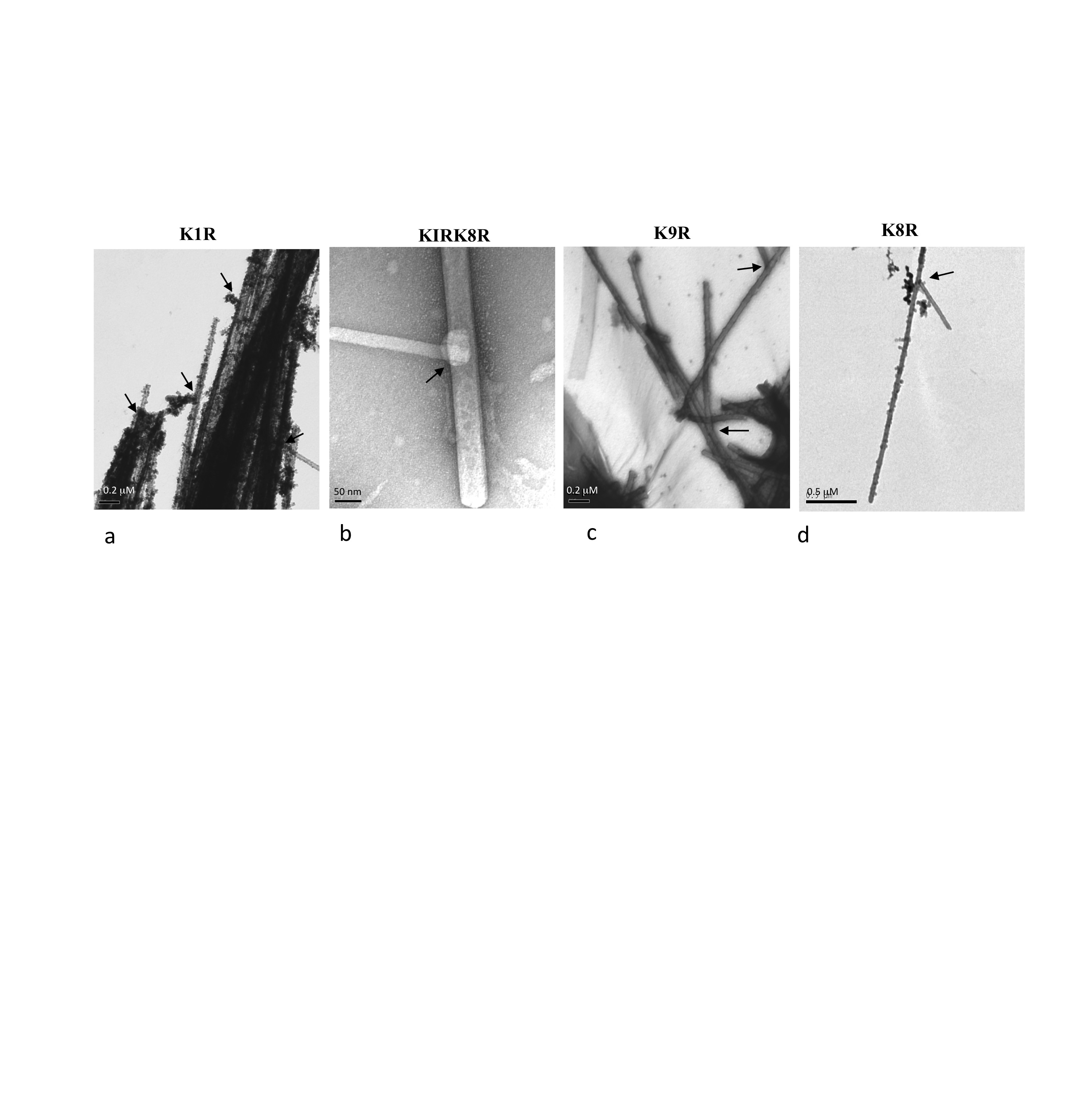


Figure S2. TEM images showing the branching of K/R- silica-NWs after one month incubation in organic solvents. a) 2-propanol, b) & c) ethanol, and d) AcN, d). Arrows indicate the branching positions.


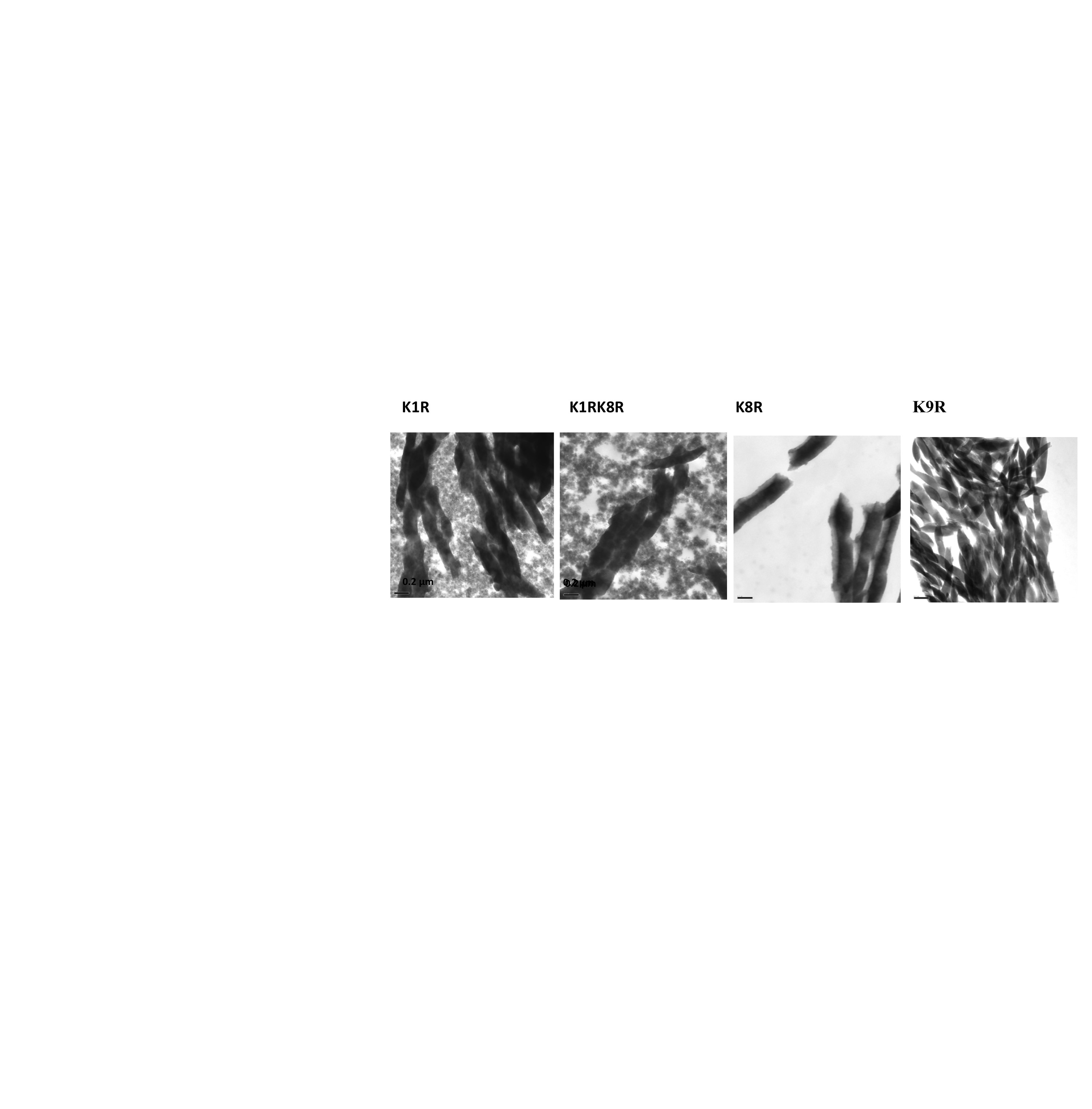


Figure S3. TEM of sectioned silica-NW after 5 months. The silica-NWs converted to thick and glass-like structures.
